# The financial burden of cancer: A longitudinal analysis of out-of-pocket healthcare costs in Australia (2009–2021)

**DOI:** 10.1007/s00520-026-11007-z

**Published:** 2026-08-08

**Authors:** Mohammad Afshar Ali, Christine Y. Lu

**Affiliations:** 1 School of Pharmacy, Faculty of Medicine and Health, The University of Sydney, Sydney, NSW 2050 Australia; 2 School of Business, Melbourne Institute of Technology, Sydney, NSW 2000 Australia; 3 School of Accounting, Southern Cross Institute, Sydney, NSW 2150 Australia; 4 Kolling Institute, The University of Sydney Faculty of Medicine and Health, and the Northern Sydney Local Health District, Sydney, NSW 2065 Australia

**Keywords:** Cancer, Out-of-pocket cost, Catastrophic healthcare expenditures, Australia

## Abstract

**Background:**

Cancer can impose substantial financial strain on patients and their families, particularly through high out-of-pocket healthcare costs. This study aims to compare out-of-pocket and catastrophic healthcare expenditures among individuals with cancer, those with non-cancer chronic conditions, and healthy individuals, and to identify socio-demographic and health-related factors associated with higher financial burden.

**Methods:**

We analysed data from four waves of the Household, Income and Labour Dynamics in Australia (HILDA) survey. Two-part and random-effects logistic regression models were used to examine associations between cancer status and total out-of-pocket and catastrophic healthcare expenditures, adjusting for socio-demographic characteristics and health service utilisation.

**Results:**

Cancer patients had significantly higher financial burden than both healthy individuals and those with non-cancer chronic conditions. Compared to healthy individuals, cancer patients had 15% higher out-of-pocket healthcare expenditures (95% CI: 1.09–1.22) and 1.05 times higher odds of experiencing catastrophic health expenditure (95% CI: 1.03–1.06). When compared to individuals with non-cancer chronic conditions, cancer patients had 11% higher out-of-pocket healthcare expenditure (95% CI: 1.06–1.17) and 1.03 times higher odds of experiencing catastrophic spending (95% CI: 1.01–1.05). Key factors associated with higher odds of catastrophic healthcare expenditure included older age, long-term disability, lower household income, and higher education attainment.

**Conclusion:**

Cancer patients had higher out-of-pocket healthcare costs and higher odds of catastrophic healthcare expenditure than comparison groups. These findings highlight the need for targeted supportive care strategies and policy interventions to reduce the financial burden associated with cancer and promote equitable access to care.

**Supplementary information:**

The online version contains supplementary material available at https://doi.org/10.1007/s00520-026-11007-z.

## Introduction

Cancer is a leading global health concern, not only due to its high mortality but also because of the complexity of care and associated long-term treatment costs. By 2030, more than 22 million people worldwide are expected to be diagnosed with cancer annually [1]. Unlike many acute illnesses, cancer often requires prolonged and intensive treatment, placing considerable financial demands on patients, families, and healthcare systems [2, 3]. These financial pressures are increasingly recognised as part of the broader supportive care burden faced by people living with cancer [4].

Globally, households affected by chronic illnesses, including cancer, face considerable out-of-pocket healthcare costs [5, 6]. In high-income countries, these households spend an estimated 16% of their annual income on care, while in low-income countries the figure is 42% [7]. When healthcare expenses exceed a household’s ability to pay, commonly defined as more than 10% of income, they are considered catastrophic [8, 9]. Around 150 million people worldwide experience catastrophic health expenditure each year, with the greatest burden observed in low- and middle-income settings [3, 8, 9], although the issue is increasingly evident in high-income countries as well.

In Australia, where healthcare is partially subsidised through public insurance, individuals still contribute significantly to their medical expenses [6, 7, 10, 11]. In 2022–23, Australians paid 15.4% of total healthcare expenditure out-of-pocket, averaging AU$1,476 per person [12]. These costs can limit access to care: nearly one in four Australians with chronic illness report delaying or avoiding care due to cost [13], and cost-related avoidance of primary care physician visits has increased from 5.0% in 2014–15 to 8.8% in 2023–24 [14]. Financial hardship is especially pronounced among socioeconomically disadvantaged groups; between 2006 and 2014, nearly 30% of individuals in the lowest income decile experienced catastrophic health expenditures [8]. These trends raise concerns about the affordability and equity of healthcare access in Australia [13, 15–17].

While international studies have increasingly documented the financial burden of cancer care [18, 19], Australian evidence remains limited. Existing studies have largely focused on specific chronic illnesses [6, 20] or population subgroups [6, 7, 17, 21, 22], rather than on cancer patients specifically. Although nearly half of cancer patients in Australia report annual out-of-pocket costs exceeding AU$5,000 [23], the extent to which these expenses exceed affordability thresholds remains unclear. Furthermore, most previous studies have used cross-sectional data [11, 24], limiting insight into changes in financial burden over time and variation between individuals.

This study builds on the existing literature by using longitudinal data from four waves of the nationally representative Household, Income and Labour Dynamics in Australia (HILDA) survey. While previous Australian studies have examined the economic and financial burden of cancer, evidence using HILDA to assess catastrophic health expenditure among people with cancer remains scarce. We compare out-of-pocket and catastrophic healthcare expenditures between individuals with cancer and two distinct reference groups: those without chronic illnesses (referred to as ‘healthy’ individuals) and those with non-cancer chronic illnesses. This dual-comparator design allows us to distinguish the financial burden experienced by people with cancer from that experienced by individuals with other chronic conditions, rather than relying on a single combined reference group. Our findings contribute to understanding financial hardship as a component of supportive cancer care and may inform patient-centred interventions and policy responses aimed at reducing economic vulnerability.

## Methods

### Healthcare system of Australia

The Australian healthcare system is a hybrid of public and private sectors, both contributing to the funding and delivery of services [25]. At its centre is Medicare, a publicly funded universal health insurance scheme designed to reduce financial barriers by subsidising medical services and prescription medicines [26]. Medicare provides free treatment in public hospitals and subsidizes out-of-hospital care through rebates based on a schedule of fees [25]. While public hospitals are state-run, most out-of-hospital care is delivered by private providers who set their own fees. Patients are responsible for covering any gap between provider charges and the Medicare rebate, resulting in out-of-pocket costs [8]. To offer additional financial protection, the Medicare Safety Net provides extra rebates to individuals and families once they exceed a specified annual threshold of out-of-pocket spending. Health Care Cards are also issued to eligible welfare recipients and low-income earners, reducing their out-of-pocket costs for prescription medications under the Pharmaceutical Benefits Scheme [27].

### Data source

This study uses data from the Household, Income and Labour Dynamics in Australia (HILDA) Survey, a nationally representative longitudinal study of Australian households. Since 2001, HILDA has annually tracked individuals aged 15 and over, collecting data on a wide range of life aspects, including household composition, income and employment, health and well-being, education and skills, family dynamics, housing, and life events. Data are collected through a combination of interviews and self-completion questionnaires. Ethical approval and data collection procedures adhere to guidelines established by the University of Melbourne, ensuring participant confidentiality and research integrity. Further details on survey’s design, sampling, and methodology can be found elsewhere [28].

### Analytic sample and missing data

**Fig. 1:**
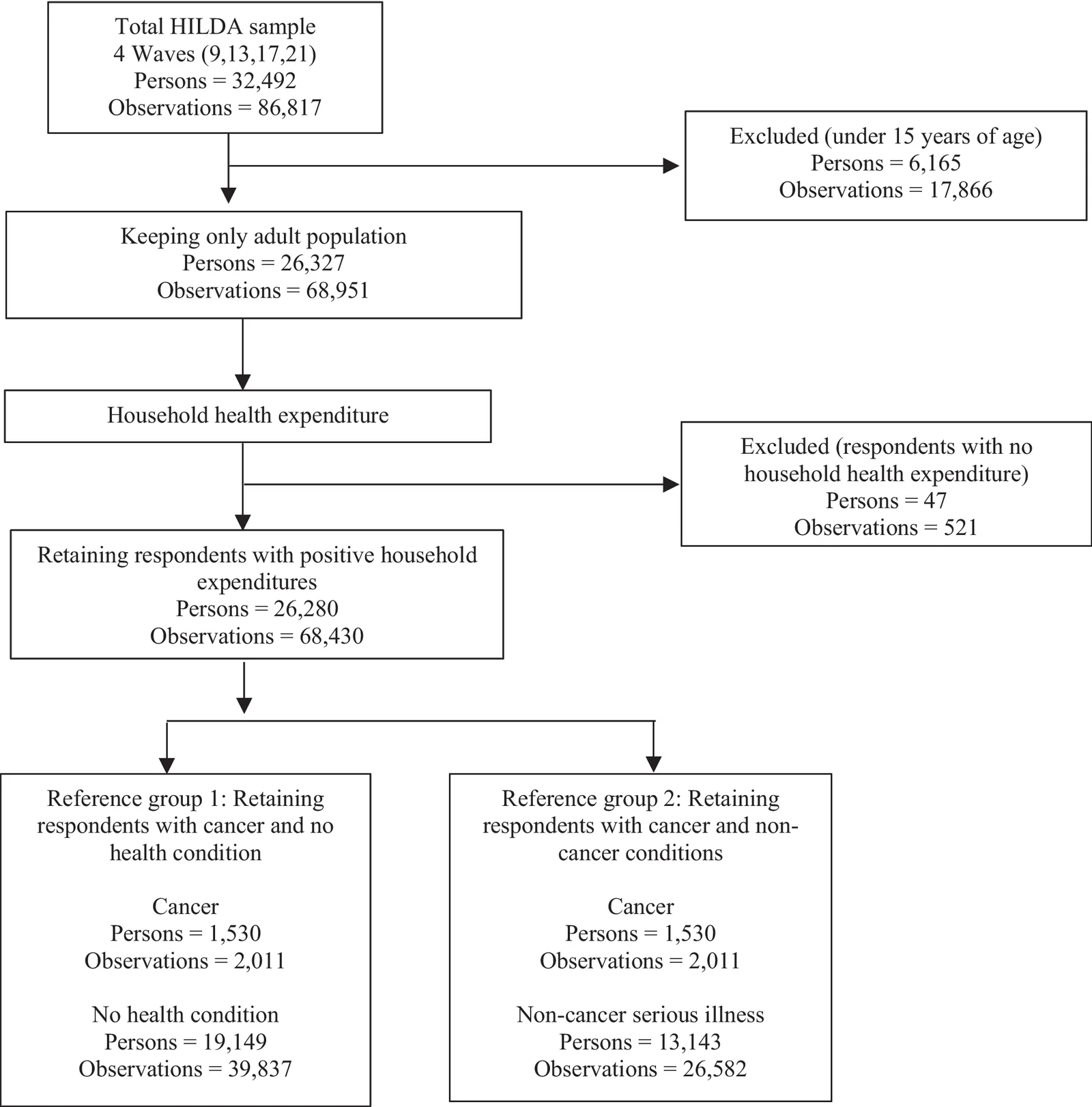
Participants flow into the analytic sample and missing data

We analysed data from Waves 9 (2009), 13 (2013), 17 (2017), and 21 (2021) of the HILDA survey. These waves were selected because they include the comprehensive health module, administered every four years since Wave 9. To reduce bias, the analysis was restricted to individuals with complete data on equivalised household income and catastrophic healthcare expenditure. Unbalanced panel datasets were constructed, allowing for variation in respondent participation across survey waves. The final pooled sample included 68,430 person-year observations from 26,280 unique individuals, stratified into three groups: (1) individuals with cancer (2,011 yearly observations from 1,530 individuals), (2) reference group 1: those without any chronic conditions (39,837 yearly observations from 19,149 individuals), and (3) reference group 2: individuals with non-cancer health conditions (26,582 yearly observations from 13,143 individuals). Figure 1 presents the flow diagram of sample construction and the treatment of missing data. Further details on data integration across waves are provided in Supplementary Table A1.

### Measures

#### Dependent variables

This study examined two measures of healthcare-related financial burden: (1) total household healthcare expenditure and (2) catastrophic health expenditure. The first outcome, overall household healthcare expenditure was measured using self-reported data on household-level spending over the previous 12 months, as captured in the HILDA study [8]. Expenditure included three categories: (i) payments to health practitioners, (ii) costs for prescriptions, pharmaceuticals, and alternative medicines, and (iii) premiums paid for private health insurance. All expenditures were reported in Australian dollars (AU$). A key limitation of the HILDA dataset is that healthcare expenditure is reported at the household level, making it difficult to isolate costs attributable solely to the respondent. Moreover, in approximately 80% of households, only one individual completes the full health module in a given wave, limiting the ability to adjust for the health status of other household members. Despite this, using household-level expenditure to examine financial burden at the individual level is consistent with prior research in both Australian and international settings [11, 29, 30]. To partially address this limitation, we included covariates for household healthcare utilisation, based on the respondent’s report of their own and their children’s number of primary care physician and hospital visits in the past 12 months. These variables serve as proxies for broader healthcare needs within the household.

The second outcome, catastrophic healthcare expenditure, was defined as household healthcare spending that accounts for 10% or more of the household’s income, consistent with previous Australian research [8]. Total household income was calculated by subtracting taxes from regular household income. To adjust for household size, household income was modified using the Organisation for Economic Co-operation and Development (OECD)-modified equivalence scale [31], which assigns weights based on the number of adults (aged 15 and over) and children (aged 14 and under) in the household. For regression modelling, catastrophic health expenditure was coded as a binary variable. All expenditure and income values were inflation-adjusted to 2021 values using the Consumer Price Index [32].

#### Key exposure variable

An individual’s cancer or serious illness status was determined using self-reported data from the HILDA survey. Respondents were asked, *"Have you ever been told by a medical practitioner that you have been diagnosed with a serious illness or medical condition?"* They were then presented with list of 11 conditions, including cancer, arthritis, asthma, chronic bronchitis or emphysema, type 1 diabetes, type 2 diabetes, heart disease, hypertension, circulatory disease, anxiety/depression, and other mental health conditions. Respondents answered ‘Yes’ or ‘No’ for each condition. Based on their responses, participants were categorized into three groups: individuals with cancer, those without any serious illness (reference group 1), and those with non-cancer serious illnesses (reference group 2). Cancer status was used as the primary exposure variable in analyses examining associations with out-of-pocket healthcare expenditure.

#### Confounders

This analysis accounted for several potential confounders associated with out-of-pocket expenditure, as identified in previous studies [11, 24]. While these factors help adjust for observed associations between cancer and healthcare costs, some relevant variables, such as cancer stage and treatment specifics, were not available in the dataset and may influence the results. Confounders included: age (15–24, 25–39, 40–64, and 65 + years), gender (male and female), relationship status (partnered and unpartnered), annual household disposable income quintile (from quintile 1 [poorest] to quintile 5 [richest]), Indigenous status (Indigenous versus non-Indigenous), region of residence (major cities versus rural and remote areas), smoking status (non-smoker versus current smoker), alcohol consumption (non-drinker versus current drinker), physical activity level (below recommended versus recommended), employment status (employed, unemployed, or not in the labor force), private health insurance coverage (yes versus no), doctor visit in the past 12 months (yes versus no), hospital admissions in the past 12 months (yes versus no), long-term disability (yes versus no; defined as an impairment, long-term health condition or disability that restricts everyday activities and has lasted, or is likely to last, for 6 months or longer), and health-related quality of life (HRQoL) measured by the SF-36 general health score (transformed from raw scale scores to a 0–100 scale). Additional information about confounder construction is provided in Supplementary Table A1.

### Statistical analyses

The analysis was conducted in two stages. First, we summarized participant characteristics using descriptive statistics. Continuous variables were presented as means, standard deviations (SD), medians and interquartile ranges (IQR), while categorical variables were reported as frequencies and percentages. Second, we explored bivariate relationships between outcome variables and cancer status. We reported mean and median healthcare expenditure and the proportion of respondents experiencing catastrophic healthcare expenditure by cancer status. Differences in median expenditure across groups were assessed using the Kruskal–Wallis rank sum test, while differences in proportions of catastrophic expenditure were evaluated with Pearson’s chi-square (χ^2^) test.

Due to the skewed distribution and the high proportion of zero values in healthcare expenditure (the first outcome variable), a two-part model was used [33, 34]. The first part used longitudinal logistic regression to estimate the likelihood of incurring any out-of-pocket healthcare costs. The second part applied a generalized linear model (GLM) with a log link and gamma distribution to model positive expenditures to assess the magnitude of spending. A separate longitudinal random-effects logistic regression model assessed the relationship between cancer and catastrophic healthcare expenditure. As an additional sensitivity analysis, we estimated individual fixed-effects linear regression models to account for unobserved time-invariant individual characteristics that may be associated with both cancer status and healthcare expenditure. The fixed-effects estimates were compared with the main random-effects results to assess the robustness of the estimated associations. Results are reported as adjusted odds ratios (aOR) or exponentiated coefficients (exp[β]), with 95% confidence intervals (CI). Statistical significance was set at p-value < 0.05.

All statistical analyses were performed using R version 4.3.1 (R Foundation for Statistical Computing, Vienna, Austria). Models were run separately for each reference group, as described above.

## Results

### Descriptive statistics

Table 1 presents data from 68,430 observations: 2.94% (n = 2,011) were individuals living with cancer, 58.22% (n = 39,837) reported no serious illness, and 38.85% (*n *= 26,582) had non-cancer serious illnesses. Table 1 also details socio-demographic, health, and employment characteristics of the analytic sample, stratified by cohort.

**Table 1:** Distribution of the analytic sample (sociodemographic characteristics, health and health care utilisation) by cohort

Variable	Reference group 1: No serious illness (*n* = 39,837)		Reference group 2: Non-cancer serious illness (*n* = 26,582)		Cancer (*n* = 2,011)	
	*n*	%	*n*	%	*n*	%
Age						
15–24	9365	23.51	2662	10.01	19	0.94
25–39	13,350	33.51	4514	16.98	117	5.82
40–64	14,359	36.04	11,013	41.43	792	39.38
65 and above	2763	6.94	8393	31.57	1083	53.85
Gender						
Male	20,454	51.34	11,419	42.96	1083	53.85
Female	19,383	48.66	15,163	57.04	928	46.15
Relationship status						
Partnered	21,152	53.10	15,335	57.69	1227	61.01
Unpartnered	18,685	46.90	11,247	42.31	784	38.99
Highest education level						
Year 12 and below	18,658	46.84	12,319	46.34	864	42.96
Professional qualifications	12,928	32.45	10,081	37.92	834	41.47
University qualifications	8251	20.71	4182	15.73	313	15.56
Equivalised household income						
Q1 (poorest)	5685	14.27	7315	27.52	685	34.06
Q2	7735	19.42	5509	20.72	442	21.98
Q3	8614	21.62	4766	17.93	307	15.27
Q4	8854	22.23	4545	17.10	286	14.22
Q5 (richest)	8948	22.46	4447	16.73	291	14.47
Indigenous status						
Not of Indigenous origin	39,730	99.73	26,497	99.68	2007	99.80
Indigenous origin	107	0.27	85	0.32	4	0.20
Region of residence						
Major city	27,474	68.97	17,000	63.95	1264	62.85
Regional city and remote area	12,363	31.03	9582	36.05	747	37.15
Disability						
Yes	5330	13.38	13,900	52.29	1287	64.00
No	34,507	86.62	12,682	47.71	724	36.00
Smoking status						
Non-smoker	34,407	86.37	22,399	84.26	1771	88.07
Current smoker	5430	13.63	4183	15.74	240	11.93
Alcohol consumption						
Non-drinker	7232	18.15	5703	21.45	449	22.33
Current drinker	32,605	81.85	20,879	78.55	1562	77.67
Physical activity						
Less than the recommended level	25,171	63.18	18,398	69.21	1401	69.67
Recommended level	14,666	36.82	8184	30.79	610	30.33
Employment status						
Employed	27,252	68.41	12,968	48.78	654	32.52
Unemployed	1437	3.61	1027	3.86	24	1.19
Not in labour force	11,148	27.98	12,587	47.35	1333	66.29
Private health insurance						
No	15,915	39.95	12,646	47.57	857	42.62
Yes	19,617	49.24	13,851	52.11	1151	57.24
Unknown/no response/not applicable	4305	10.81	85	0.32	3	0.15
Number of family doctor or GP visits (mean, SD)	5.08 (6.88)		8.21 (9.49)		10.40 (11.30)	
Number of hospital admissions (mean, SD)	0.20 (0.92)		0.37 (1.38)		0.77 (1.40)	
Health related quality of life (HRQoL)						
SF-36 general health (mean, SD)	74.10 (71.20)		60.10 (21.50)		52.20 (23.80)	

Most cancer patients were older adults (53.85%), partnered (53.85%), non-Indigenous (99.80%), and resided in major cities (62.85%). Furthermore, 64.00% reported having a long-term disability, 11.93% were current smokers, 77.67% consumed alcohol, and 69.67% were physically inactive. Notably, 66.29% were out of the labour force, compared to 27.98% and 47.35% in the no-illness and non-cancer serious illness groups. The average health-related quality of life (HRQoL) score (SF-36) in the cancer group was 52.20 (standard deviation [SD] = 23.80). Most (57.24%) had private health insurance. On average, cancer patients and their households had 10.40 visits to a primary care physician (SD = 11.30) and 0.77 hospital admissions (SD = 1.40) in the past year.

The Anderson–Darling normality test confirmed that the primary outcome variable, total healthcare expenditure, was not normally distributed (statistic = 3742.80, *p* < 0.001). The distribution was positively skewed (skewness = 4.42), with many low or zero values and a few extremely high costs (see Figure A1).

Table 2 compares healthcare expenditures (AU$) and catastrophic health expenditure prevalence across three study groups. Average expenditure was AU$2705.46 (SD = AU$3365.38) for those without serious illness, AU$2913.79 (SD = AU$3539.22) for those with non-cancer conditions, and highest among cancer patients at AU$3502.26 (SD = AU$3851.88). Median expenditure was also significantly higher for cancer patients (AU$1780.00; IQR = AU$3865.50) compared to others (Kruskal–Wallis rank sum test statistic, p < 0.001). Additionally, catastrophic healthcare expenditure affected 14.36% of those without serious illness, 21.65% with non-cancer serious illnesses, and 30.58% of cancer patients.

**Table 2:** Out-of-pocket healthcare expenditures by cohort

Outcome	No serious illness		Non-cancer serious illness		Cancer		Significance
	Mean (SD)	Median (IQR)	Mean (SD)	Median (IQR)	Mean (SD)	Median (IQR)	*p* value (Kruskal–Wallis rank sum test)
Total healthcare expenditure (AU$)	2705.46 (3365.38)	1690.00 (3600.00)	2913.79 (3539.22)	1780.00 (3865.50)	3502.26 (3851.88)	2460.00 (4289.50)	< 0.001
Household expenditure on healthcare professionals (AU$)	946.40 (2120.15)	400.00 (950.00)	968.59 (2112.32)	360.00 (1000.00)	1268.50 (2439.35)	500.00 (1132.50)	< 0.001
Household expenditure on medication (AU$)	374.11 (649.47)	200.00 (450.00)	549.02 (793.60)	312.50 (580.00)	636.47 (978.74)	400.00 (650.00)	< 0.001
Household expenditure on private health insurance (AU$)	1384.94 (1776.91)	660.00 (2425.00)	1396.18 (1808.18)	530.00 (2460.00)	1597.28 (1814.95)	1052.50 (2756.00)	< 0.001
Proportion with catastrophic health expenditure (%)	*n*	%	*n*	%	*n*	%	*p* value (Chi-squared test)
No	34,118	85.64	20,826	78.35	1396	69.42	< 0.001
Yes	5719	14.36	5756	21.65	615	30.58	

### Regression modelling

**Fig. 2:**
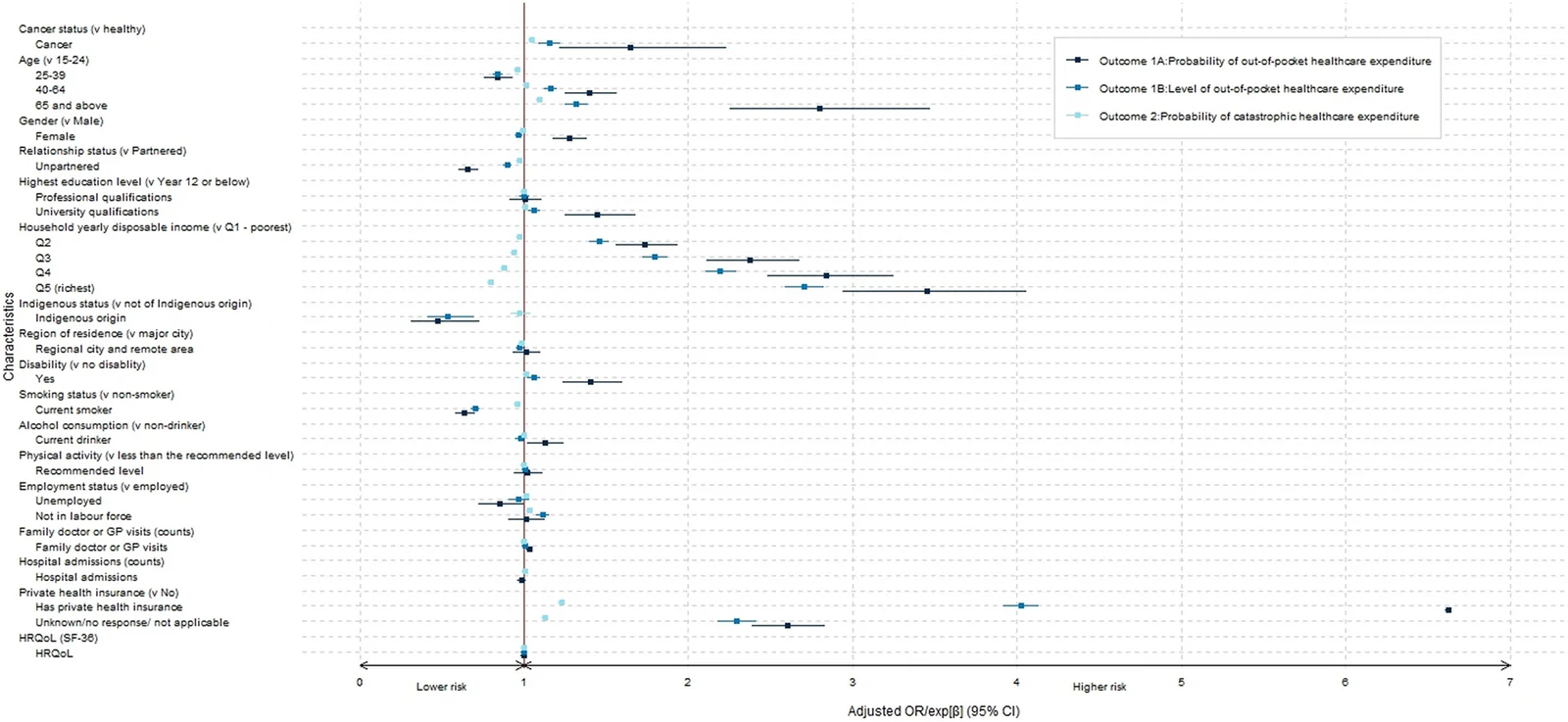
Results on out-of-pocket healthcare expenditure from two-part and random-effects logistic regression (individuals with cancer vs. those without serious illness)

**Fig. 3:**
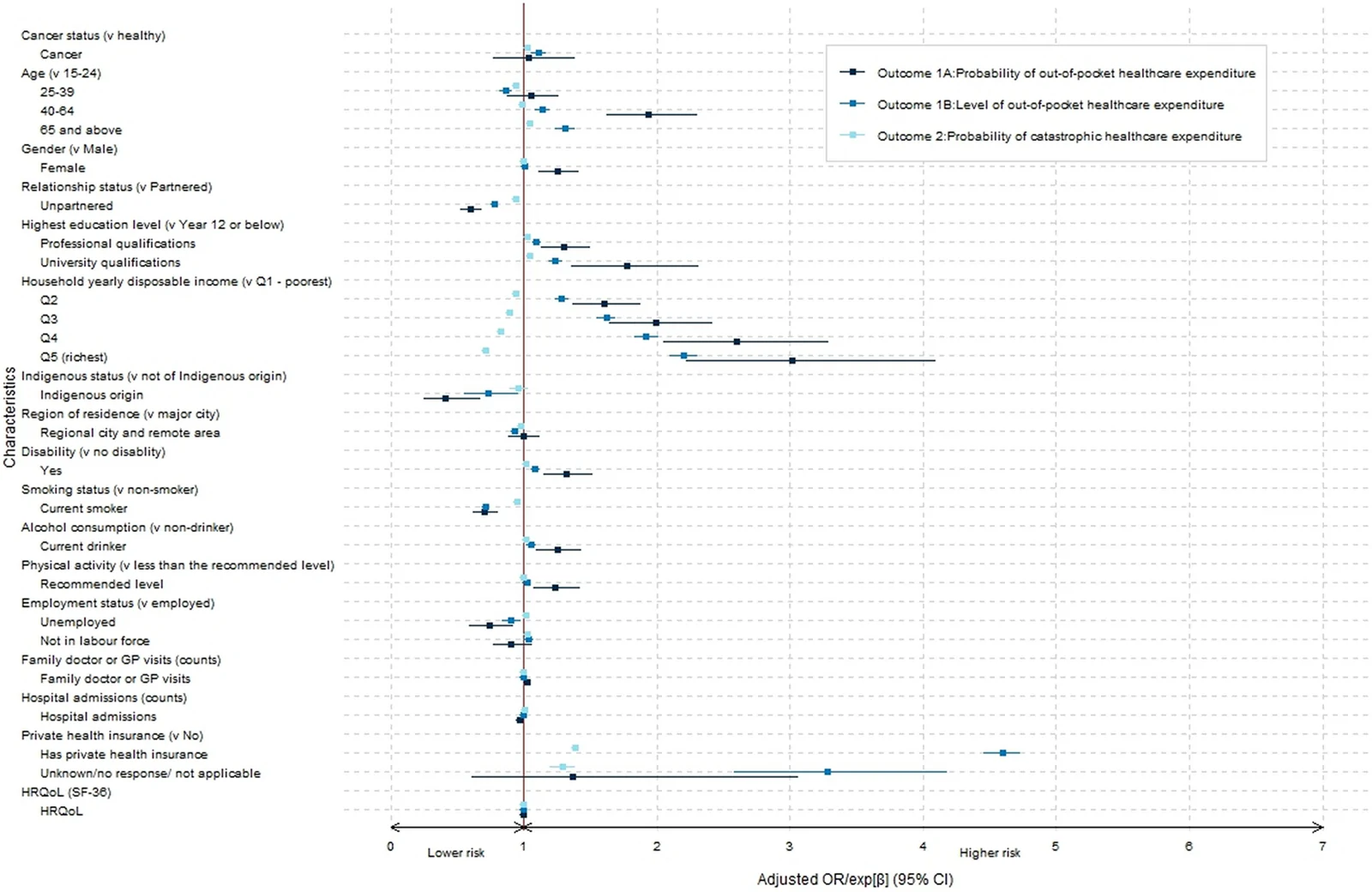
Results on out-of-pocket healthcare expenditure from two-part and random-effects logistic regression (individuals with cancer vs. with non-cancer serious illness)

Figures 2 and 3 present adjusted regression results comparing cancer patients to two distinct reference groups: individuals without serious illness (41,848 person-year observations) and those with non-cancer health conditions (28,593 person-year observations). Outcomes assessed were total healthcare expenditure (Outcome 1) and catastrophic health expenditure (Outcome 2).

After adjusting for sociodemographic, health, job-related, and healthcare utilisation covariates, individuals with cancer had 65% higher odds of any out-of-pocket expenditure (aOR: 1.65; 95% CI: 1.21–2.23), and among spenders, costs were 15% higher (exp(β): 1.15; 95% CI: 1.09–1.22) than those without serious illness (Fig. 2, Supplementary Table A4, Model 1 A and 1B). When compared to those with non-cancer illnesses, cancer patients with positive spending had 11% higher costs (exp(β): 1.11; 95% CI: 1.06–1.17) (Fig. 2, Supplementary Table A5, Model 1B).

Cancer was also linked to a modest but statistically significant increase in catastrophic health expenditure odds: 5% higher versus no serious illness (aOR: 1.05; 95% CI: 1.03–1.06; Model 2) (Fig. 2, Supplementary Table A4, Model 2) and 3% higher versus non-cancer illnesses (aOR: 1.03; 95% CI: 1.01–1.05) (Fig. 3, Supplementary Table A5, Model 2).

When comparing individuals with cancer to those without serious illness (reference group 1), several factors were significantly associated with higher odds of catastrophic healthcare expenditure. These included older age (65 and above) (aOR: 1.09; 95% CI: 1.08–1.11), and higher education (university qualifications) (aOR: 1.01; 95% CI: 1.00–1.02). Conversely, being unpartnered (aOR: 0.97; 95% CI: 0.97–0.98) and belonging to a high-income household (aOR: 0.80; 95% CI: 0.79–0.81) were associated with lower odds of catastrophic healthcare expenditure. Notably, private health insurance was linked to increased likelihood of experiencing catastrophic healthcare expenditure (aOR: 1.23; 95% CI: 1.22–1.24). Similar patterns were observed when comparing individuals with cancer to those with non-cancer serious illnesses (reference group 2). Estimates from the sensitivity analysis using individual fixed-effects models were used to account for unobserved time-invariant individual characteristics. The fixed-effects results were consistent with the main random-effects findings (Supplementary Table A6), showing a statistically significant association between cancer and higher healthcare expenditure. These findings are consistent with the main results. The determinants of total healthcare expenditure closely mirrored those of catastrophic healthcare expenditure, suggesting the heightened financial vulnerability of individuals with cancer.

## Discussion

This study is among the first in Australia to use nationally representative longitudinal data to quantify both out-of-pocket and catastrophic healthcare spending, defined as spending exceeding 10% of income, specifically for individuals with cancer, and to compare them with people without serious illness and those living with non-cancer chronic conditions.

Our findings reveal that cancer patients face a substantially higher financial burden. They incur an average AU$3,502 per year in household healthcare expenditures, considerably more than the AU$2,750 for those without serious illness and AU$2,914 for those with other chronic conditions. These figures notably exceed the average out-of-pocket spending reported by the Australian Institute of Health and Welfare (AU$1,476) [12], but are consistent with previous HILDA-based estimates of about AU$3,100 (2006–2014) [8]. This elevated cost burden is consistent with earlier studies, including one study that reported median two-year out-of-pocket expenses for cancer patients between AU$1,078 and AU$4,192, compared with just AU$615 for the general population [7]. Collectively, these findings highlight the disproportionate financial strain associated with cancer, even within Australia’s universal healthcare system.

Importantly, 30.6% of cancer patients in our study experienced catastrophic health expenditure, markedly higher than the rates observed among individuals with non-cancer chronic conditions (21.7%) and people without serious illness (14.4%). This rate also exceeds estimates from other high-income settings, including the United States (27.6%) [35] and an average of 23.4% across several developed countries [5].

Interestingly, while cancer patients were more likely to spend and spend more, the relative increase in catastrophic spending was modest, highlighting that financial vulnerability depends not only on expenditure level but also household resilience. Wealthier households spent more but were less likely to cross the catastrophic threshold, while lower-income households faced higher vulnerability even with modest spending. These disparities highlight persistent inequities in health-related financial risk [24, 29, 36].

Several sociodemographic and health-related factors were associated with higher financial burden. Older age (65 +), long-term disability, and university-level education all increased the odds of catastrophic spending, possibly reflecting greater healthcare utilization or more proactive engagement with health services. Women incurred higher costs than men, consistent with broader patterns in healthcare-seeking behavior [37, 38]. Being unpartnered was linked with lower risk of catastrophic spending, potentially reflecting reduced care-seeking due to limited support.

Regional and socioeconomic differences also influenced financial outcomes. Residents in rural or remote areas had slightly lower odds of catastrophic health spending, which may reflect reduced healthcare utilisation rather than better financial protection, raising concerns about underuse of essential services in these regions [36]. This may also be linked to lower educational attainment in disadvantaged areas, which can reduce health awareness and engagement with care [39]. Notably, private health insurance was associated with higher odds of catastrophic spending, reflecting that insurance coverage does not necessarily guarantee financial protection, particularly in the context of cost-sharing arrangements such as copayments and deductibles [24, 40–42].

These findings have important policy implications because our results show that individuals with cancer experience higher out-of-pocket healthcare expenditure and a greater risk of catastrophic health expenditure compared with both individuals without chronic illness and those with non-cancer chronic conditions. This suggests that existing financial protection mechanisms may not be sufficient to prevent cancer-related financial hardship, even within the Australian healthcare system. Policy responses should therefore focus on reducing the direct cost burden faced by cancer patients, particularly through better targeted subsidies, income-sensitive out-of-pocket cost caps, and support mechanisms for patients at higher risk of catastrophic expenditure.

Finally, the consistent predictors across both comparison groups are consistent with the relevance of these findings beyond Australia. High-income countries grappling with the economic impact of cancer care can draw on this evidence to inform targeted, equity-oriented policies that support those most vulnerable.

This study has several limitations. First, it relies on self-reported healthcare expenditure, which may be affected by recall bias. Nonetheless, this approach is consistent with prior Australian research using individual-level survey data to assess out-of-pocket healthcare costs [8, 11], and the expenditure patterns reported here are comparable to those found in earlier studies [8, 11]. Future research could enhance accuracy by drawing on linked administrative data sources, such as the Person Level Integrated Data Asset via the Australia Bureau of Statistics or the National Health Data Hub via the Australian Institute of Health and Welfare. Second, the analysis is limited by the use of household-level out-of-pocket healthcare expenditure to approximate individual-level financial burden, as the HILDA survey does not provide person-level expenditure data. While this is a common constraint in similar studies, it may obscure variation in costs attributable to other household members. Third, our measure of total healthcare expenditure does not capture associated indirect costs, such as transport, accommodation, or productivity losses for patients and caregivers. Including these components in future analyses would provide a more complete picture of the financial burden. Fourth, although the fixed-effects sensitivity analysis yielded estimates consistent with the main results for the association between cancer and healthcare expenditure, residual time-varying confounding may remain; therefore, findings should be interpreted as associations rather than causal effects. Finally, the analysis does not stratify by specific expenditure categories, illness types, cancer site, time since diagnosis, or disease severity, all of which can significantly affect the extent and nature of healthcare costs [24, 40]. Addressing these nuances would allow for more targeted policy and clinical responses.

## Conclusion

Out-of-pocket payments remain one of the most regressive forms of healthcare financing, disproportionately affecting those with the greatest need. This study demonstrates that individuals living with cancer face significantly higher out-of-pocket healthcare costs and a greater risk of catastrophic health expenditure than both people without serious illness and those with non-cancer chronic conditions. By using two distinct reference groups, the findings highlight the uniquely high financial burden associated with cancer. In addition to cancer status, several socio-demographic, health, and healthcare utilization factors were associated with increased financial vulnerability. These elevated costs not only intensify household financial strain but may also delay access to care, restrict treatment choices, and negatively impact health outcomes. These findings emphasize the urgent need for policy actions to alleviate the financial burden of cancer care in Australia. This includes targeted efforts to lower out-of-pocket costs, improve financial protection mechanisms, and address disparities affecting disadvantaged groups. Ensuring equitable access to affordable care is essential to supporting the broader goals of supportive cancer care.

## Supplementary Information

Below is the link to the electronic supplementary material.

ESM 1 (DOCX 106 KB)

## Data Availability

This study used data from the Household, Income and Labour Dynamics in Australia (HILDA) Survey, conducted by the Melbourne Institute of Applied Economic and Social Research (https://melbourneinstitute.unimelb.edu.au/). Although HILDA data is not publicly accessible, eligible researchers may obtain access by adhering to the Melbourne Institute’s established procedures and meeting the necessary eligibility criteria.
