# Supplementary material for: The financial burden of cancer: A longitudinal analysis of out-of-pocket healthcare costs in Australia (2009–2021)
Source: Support Care Cancer. 2026 Aug 8;34(9):835. doi: 10.1007/s00520-026-11007-z (PMC13452749; doi:10.1007/s00520-026-11007-z)
Supplement: Supplementary file 1 [file 520_2026_11007_MOESM1_ESM.docx]

**Supplementary materials**

Table A1: Distribution of study analytic sample by HILDA wave.

| **Wave** | **Group** | **Frequency** |
| --- | --- | --- |
| 9 | No serious illness | 8774 |
|  | Cancer | 412 |
|  | Non–cancer serious illness | 4686 |
| 13 | No serious illness | 11623 |
|  | Cancer | 510 |
|  | Non–cancer serious illness | 6309 |
| 17 | No serious illness | 10166 |
|  | Cancer | 568 |
|  | Non–cancer serious illness | 7747 |
| 21 | No serious illness | 9274 |
|  | Cancer | 521 |
|  | Non–cancer serious illness | 7840 |

Table A2: Description of the control variables.

| **Variables** | **Measure** |
| --- | --- |
| **Socio–demographic characteristics** | |
| Age (years) | 0 = 15–24  1 = 25–39  2 = 40–64  3 = 65 and above |
| Gender | 0 = Male  1 = Female |
| Relationship status | 0 = Partnered (in a registered marriage or de facto relationship)  1 = Unpartnered (never married, separated but not divorced, divorced, or widowed) |
| Highest level of educational attainment | 0 = Year 12 and below (year 12, and year 11 and below)  1 = Professional qualification (advance diploma or diploma, and certificate III or IV)  2 = University qualification (postgraduate—masters or doctorate, graduate diploma or certificate, bachelor or honours) |
| Household yearly  disposable income | 0 = Quintile 1 (poorest)  1 = Quintile 2 (poorer)  2 = Quintile 3 (middle)  3 = Quintile 4 (richer)  4 = Quintile 5 (richest) |
| Indigenous status | 0 = Not of Indigenous origin  1 = Indigenous origin (Aboriginal, Torres Strait Islander, and both Aboriginal and Torres Strait  Islander) |
| Region of residence | 0 = Major city  1 = Regional or remote area including inner regional, outer regional, remote and very remote Australia |
| **Health–related characteristics** | |
| Weight category | 0 = Underweight (BMI less than 18.50)  1 = Healthy weight (BMI 18.50–24.99)  2 = Overweight or pre–obese (BMI 25.00–29.99)  3 = Obese (BMI 30 or greater) |
| Long–term health condition or disability | 0 = No  1 = Yes |
| HRQoL (SF–36 general health) | Health–related quality of life (HRQoL), measured by SF–36 general health score (transformed from raw scale scores) to a 0 to 100 scale. |
| **Health–related behaviours** | |
| Smoking status | 0 = Non–smoker (never smoked, and former smoker)  1 = Current smoker (smoke daily, smoke at least weekly and smoke less often than weekly) |
| Alcohol consumption | 0 = Non–drinker (never drunk and ex–drinker)  1 = Current drinker (only rarely, 1–2 days, 2–3 days, 3–4 days, 5–6 days per week and every day) |
| Physical activity | 0 = Less than the recommended level (not at all, less than once, 1–2 and 3 times a week)  1 = Recommended level (> 3 times a week and every day) |
| **Job–related characteristics** |  |
| Employment contract | 0 = Employed  1 = Unemployed  2 = Not in the labour force |
| **Health service utilisation** |  |
| Family doctor or GP visits (counts) | Total number of visits to a family doctor/GP in the past 12 months by the respondent and children in the same household |
| Hospital admissions (counts) | Total number of times admitted to a hospital in the past 12 months by the respondent and children in the same household |

Table A3: Within–group variation in descriptive statistics for the non–cancer condition group.

| **Non–cancer condition** | **N** | **Mean** | **SD** | **Median** | **IQR** |
| --- | --- | --- | --- | --- | --- |
| Anxiety | 4558 | 2606.84 | 3733.67 | 1155 | 3414.5 |
| Arthritis or osteoporosis | 10004 | 3073.78 | 3664.31 | 2000 | 3950.25 |
| Asthma | 7114 | 2699.81 | 3705.2 | 1400 | 3652.5 |
| Chronic bronchitis or emphysema | 1197 | 2233.54 | 3238.19 | 1000 | 2780 |
| Type 1 diabetes | 560 | 2649.82 | 3188.66 | 1400 | 3625.25 |
| Type 2 diabetes | 2979 | 2788.57 | 3404.52 | 1686.67 | 3559 |
| Depression | 4210 | 2542.29 | 4057.22 | 1075 | 3289.25 |
| High blood pressure or hypertension | 10505 | 3035.4 | 3471.22 | 2000 | 3881 |
| Heart disease | 2632 | 3149.73 | 3617.39 | 2000 | 4113.75 |
| Other serious circulatory condition | 1624 | 2755.33 | 3325.83 | 1600 | 3550 |
| Other mental illness | 1578 | 2166.79 | 3586.7 | 760 | 2525.75 |

Note: SD, standard deviation; IQR, interquartile range.

Table A4: Results on out-of-pocket healthcare expenditure from two-part and random-effects logistic regression (individuals with cancer vs. those without serious illness).

| **Variables** | **Model 1** | | **Model 2: Catastrophic healthcare expenditure**  **aOR**  **(95%CI)** |
| --- | --- | --- | --- |
|  | **Part A: Probability of any out–of–pocket healthcare expenditure**  **(aOR, 95% CI)** | **Part B:**  **Level of expenditure among spenders**  **(exp[β]), 95% CI)** |  |
| Intercept | 1.91*  [1.49 – 2.46] | 356.29*  [328.29 – 386.27] | 1.17*  [1.15 – 1.20] |
| **Chronic illness** |  |  |  |
| No serious illness (ref) |  |  |  |
| Cancer | 1.65*  [1.21 – 2.23] | 1.15*  [1.09 – 1.22] | 1.05*  [1.03 – 1.06] |
| **Age** |  |  |  |
| 15–24 (ref) |  |  |  |
| 25–39 | 0.84*  [0.75 – 0.93] | 0.84*  [0.81 – 0.87] | 0.96*  [0.95 – 0.97] |
| 40–64 | 1.40*  [1.25 – 1.57] | 1.16*  [1.12 – 1.20] | 1.01*  [1.00 – 1.02] |
| 65 and above | 2.80*  [2.26 – 3.48] | 1.32*  [1.25 – 1.39] | 1.09*  [1.08 – 1.11] |
| **Gender** |  |  |  |
| Male (ref) |  |  |  |
| Female | 1.27*  [1.17 – 1.38] | 0.97*  [0.95 – 0.99] | 0.99*  [0.98 – 1.00] |
| **Relationship status** |  |  |  |
| Partnered (ref) |  |  |  |
| Unpartnered | 0.66*  [0.60 – 0.72] | 0.90*  [0.87 – 0.92] | 0.97*  [0.97 – 0.98] |
| **Highest education level** |  |  |  |
| Year 12 and below (ref) |  |  |  |
| Professional qualifications | 1.01  [0.91 – 1.11] | 0.99  [0.97 – 1.03] | 1.00  [0.99 – 1.01] |
| University qualifications | 1.45*  [1.25 – 1.68] | 1.06*  [1.02 – 1.10] | 1.01*  [1.00 – 1.02] |
| **Household yearly disposable income** |  |  |  |
| Q1 (poorest) (ref) |  |  |  |
| Q2 | 1.74*  [1.56 – 1.94] | 1.46*  [1.40 – 1.52] | 0.97*  [0.96 – 0.98] |
| Q3 | 2.38*  [2.11 – 2.68] | 1.80*  [1.72 – 1.87] | 0.94*  [0.93 – 0.95] |
| Q4 | 2.84*  [2.48 – 3.25] | 2.19*  [2.10 – 2.29] | 0.88*  [0.87 – 0.89] |
| Q5 (richest) | 3.46*  [2.94 – 4.06] | 2.71*  [2.59 – 2.83] | 0.80*  [0.79 – 0.81] |
| **Indigenous status** |  |  |  |
| Not of Indigenous origin (ref) |  |  |  |
| Indigenous origin | 0.48*  [0.31 – 0.73] | 0.54*  [0.41 – 0.69] | 0.98  [0.92 – 1.04] |
| **Region of residence** |  |  |  |
| Major city (ref) |  |  |  |
| Regional city and remote area | 1.02  [0.93 – 1.10] | 0.98  [0.95 – 1.00] | 0.99*  [0.98 – 1.00] |
| **Long–term condition or disability** |  |  |  |
| No (ref) |  |  |  |
| Yes | 1.41*  [1.24 – 1.60] | 1.06*  [1.02 – 1.10] | 1.01*  [1.00 – 1.02] |
| **Smoking status** |  |  |  |
| Non–smoker (ref) |  |  |  |
| Current smoker | 0.64*  [0.58 – 0.70] | 0.70*  [0.68 – 0.73] | 0.96*  [0.95 – 0.97] |
| **Alcohol consumption** |  |  |  |
| Non–drinker (ref) |  |  |  |
| Current drinker | 1.13*  [1.02 – 1.24] | 0.98  [0.95 – 1.01] | 1.00  [0.99 – 1.01] |
| **Physical activity** |  |  |  |
| Less than the recommended level (ref) |  |  |  |
| Recommended level | 1.02  [0.94 – 1.11] | 1.01  [0.98 – 1.03] | 1.00  [0.99 – 1.00] |
| **Employment contract** |  |  |  |
| Employed (ref) |  |  |  |
| Unemployed | 0.85  [0.72 – 1.00] | 0.97  [0.91 – 1.04] | 1.01  [0.99 – 1.03] |
| Not in labour force | 1.01  [0.91 – 1.13] | 1.11*  [1.08 – 1.15] | 1.03*  [1.02 – 1.04] |
| **Family doctor or GP visits (counts)** | 1.03*  [1.03 – 1.04] | 1.01*  [1.01 – 1.02] | 1.01*  [1.00 – 1.01] |
| **Hospital admissions (counts)** | 0.99  [0.96 – 1.02] | 0.99  [0.98 – 1.00] | 1.00  [1.00 – 1.01] |
| **Private health insurance** |  |  |  |
| No (ref) |  |  |  |
| Yes | 6.62*  [5.85 – 7.51] | 4.02*  [3.92 – 4.13] | 1.23*  [1.22 – 1.24] |
| Unknown/no response/ not applicable | 1.83*  [1.56 – 2.14] | 2.29*  [2.18 – 2.42] | 1.13*  [1.11 – 1.15] |
| **HRQoL (SF–36 general health)** | 1.00*  [0.99 – 1.00] | 1.00*  [0.99 – 1.00] | 1.00  [0.99 – 1.00] |
| N | Persons = 20,085  Observations = 41,848 | | |

Notes: 1) 95% confidence intervals are reported in parentheses; 2) * Indicate significance at the 5% level; 3) aOR, adjusted odds ratio; exp[β], exponentiated coefficients; Ref, reference category.

Table A5: Results on out-of-pocket healthcare expenditure from two-part and random-effects logistic regression (individuals with cancer vs. with non-cancer serious illness).

| **Variables** | **Model 1** | | **Model 2: Catastrophic healthcare expenditure**  **aOR**  **(95%CI)** |
| --- | --- | --- | --- |
|  | **Part A: Probability of any out–of–pocket healthcare expenditure**  **(aOR, 95% CI)** | **Part B:**  **Level of expenditure among spenders**  **(exp[β]), 95% CI)** |  |
| Intercept | 3.17*  [2.18 – 4.61] | 408.97*  [372.68 – 448.80] | 1.20*  [1.17 – 1.24] |
| **Chronic illness** |  |  |  |
| Non–cancer serious illness (ref) |  |  |  |
| Cancer | 1.03  [0.77 – 1.39] | 1.11*  [1.06 – 1.17] | 1.03*  [1.01 – 1.05] |
| **Age** |  |  |  |
| 15–24 (ref) |  |  |  |
| 25–39 | 1.05  [0.88 – 1.26] | 0.87*  [0.82 – 0.92] | 0.94*  [0.93 – 0.96] |
| 40–64 | 1.93*  [1.62 – 2.30] | 1.14*  [1.08 – 1.20] | 0.99  [0.97 – 1.01] |
| 65 and above | 4.73*  [3.79 – 5.91] | 1.31*  [1.23 – 1.38] | 1.04*  [1.02 – 1.06] |
| **Gender** |  |  |  |
| Male (ref) |  |  |  |
| Female | 1.25*  [1.11 – 1.42] | 1.01  [0.98 – 1.03] | 1.00  [0.99 – 1.01] |
| **Relationship status** |  |  |  |
| Partnered (ref) |  |  |  |
| Unpartnered | 0.60*  [0.53 – 0.68] | 0.78*  [0.76 – 0.80] | 0.94*  [0.93 – 0.95] |
| **Highest education level** |  |  |  |
| Year 12 and below (ref) |  |  |  |
| Professional qualifications | 1.30*  [1.14 – 1.50] | 1.10*  [1.06 – 1.13] | 1.02*  [1.01 – 1.03] |
| University qualifications | 1.77*  [1.36 – 2.32] | 1.24*  [1.19 – 1.29] | 1.05*  [1.03 – 1.06] |
| **Household yearly disposable income** |  |  |  |
| Q1 (poorest) (ref) |  |  |  |
| Q2 | 1.60*  [1.37 – 1.88] | 1.29*  [1.24 – 1.34] | 0.94*  [0.93 – 0.95] |
| Q3 | 2.00*  [1.65 – 2.42] | 1.62*  [1.55 – 1.69] | 0.89*  [0.88 – 0.91] |
| Q4 | 2.60*  [2.05 – 3.29] | 1.92*  [1.83 – 2.01] | 0.82*  [0.81 – 0.84] |
| Q5 (richest) | 3.02*  [2.22 – 4.10] | 2.20*  [2.09 – 2.31] | 0.71*  [0.70 – 0.73] |
| **Indigenous status** |  |  |  |
| Not of Indigenous origin (ref) |  |  |  |
| Indigenous origin | 0.41*  [0.25 – 0.67] | 0.73*  [0.56 – 0.96] | 0.96  [0.89 – 1.04] |
| **Region of residence** |  |  |  |
| Major city (ref) |  |  |  |
| Regional city and remote area | 1.00  [0.88 – 1.13] | 0.94*  [0.91 – 0.96] | 0.98*  [0.97 – 0.99] |
| **Long–term condition or disability** |  |  |  |
| No (ref) |  |  |  |
| Yes | 1.32*  [1.15 – 1.52] | 1.08*  [1.05 – 1.12] | 1.02*  [1.01 – 1.03] |
| **Smoking status** |  |  |  |
| Non–smoker (ref) |  |  |  |
| Current smoker | 0.71*  [0.62 – 0.81] | 0.71*  [0.68 – 0.74] | 0.95*  [0.94 – 0.97] |
| **Alcohol consumption** |  |  |  |
| Non–drinker (ref) |  |  |  |
| Current drinker | 1.25*  [1.09 – 1.44] | 1.05*  [1.02 – 1.09] | 1.02*  [1.01 – 1.03] |
| **Physical activity** |  |  |  |
| Less than the recommended level (ref) |  |  |  |
| Recommended level | 1.24*  [1.07 – 1.42] | 1.02  [0.99 – 1.05] | 1.00  [0.99 – 1.01] |
| **Employment contract** |  |  |  |
| Employed (ref) |  |  |  |
| Unemployed | 0.74*  [0.59 – 0.92] | 0.90*  [0.84 – 0.98] | 1.02  [0.99 – 1.04] |
| Not in labour force | 0.90  [0.77 – 1.06] | 1.04  [1.00 – 1.07] | 1.03*  [1.01 – 1.04] |
| **Family doctor or GP visits (counts)** | 1.02*  [1.01 – 1.03] | 1.00*  [0.99 – 1.00] | 1.01  [0.99 – 1.01] |
| **Hospital admissions (counts)** | 0.97*  [0.95 – 1.00] | 1.00  [0.99 – 1.01] | 1.01*  [1.00 – 1.01] |
| **Private health insurance** |  |  |  |
| No (ref) |  |  |  |
| Yes | 7.46*  [6.00 – 9.28] | 4.59*  [4.46 – 4.73] | 1.38*  [1.37 – 1.40] |
| Unknown/no response/ not applicable | 1.37  [0.61 – 3.06] | 3.28*  [2.58 – 4.18] | 1.29*  [1.19 – 1.39] |
| **HRQoL (SF–36 general health)** | 1.00*  [0.99 – 1.00] | 1.00*  [0.99 – 1.00] | 1.00*  [0.99 – 1.00] |
| N | Persons = 13,719  Observations = 28,593 | | |

Notes: 1) 95% confidence intervals are reported in parentheses; 2) * Indicate significance at the 5% level; 3) aOR, adjusted odds ratio; exp[β], exponentiated coefficients; Ref, reference category.

Table A6: Results on out-of-pocket healthcare expenditure from fixed-effects logistic regression (individuals with cancer vs. those without serious illness, and individuals with cancer vs. with non-cancer serious illness).

| **Variables** | **Model 2: Catastrophic healthcare expenditure**  **aOR**  **(95%CI)** | |
| --- | --- | --- |
|  | **Reference group 1** | **Reference group 2** |
| **Chronic illness** |  |  |
| Non–cancer serious illness (ref) |  |  |
| Cancer | 1.05*  [1.02 – 1.08] | 1.03*  [1.01 – 1.06] |
| **Age** |  |  |
| 15–24 (ref) |  |  |
| 25–39 | 1.01  [0.99 – 1.02] | 1.03  [0.99 – 1.07] |
| 40–64 | 1.06*  [1.04 – 1.09] | 1.13*  [1.07 – 1.18] |
| 65 and above | 1.16*  [1.12 – 1.20] | 1.19*  [1.12 – 1.25] |
| **Gender** |  |  |
| Male (ref) |  |  |
| Female | 0.94  [0.63 –1.42] | 1.26  [0.58 – 2.76] |
| **Relationship status** |  |  |
| Partnered (ref) |  |  |
| Unpartnered | 0.98  [0.97 – 1.00] | 0.98*  [0.95 – 1.00] |
| **Highest education level** |  |  |
| Year 12 and below (ref) |  |  |
| Professional qualifications | 0.99  [0.98 – 1.00] | 0.99  [0.95 – 1.03] |
| University qualifications | 0.97  [0.95 – 1.00] | 0.96  [0.90 – 1.02] |
| **Household yearly disposable income** |  |  |
| Q1 (poorest) (ref) |  |  |
| Q2 | 0.95*  [0.93 – 0.96] | 0.91*  [0.89 – 0.92] |
| Q3 | 0.90*  [0.88 – 0.91] | 0.84*  [0.82 – 0.85] |
| Q4 | 0.84*  [0.83 – 0.86] | 0.76*  [0.74 – 0.77] |
| Q5 (richest) | 0.76*  [0.75 – 0.78] | 0.65*  [0.64 – 0.67] |
| **Indigenous status** |  |  |
| Not of Indigenous origin (ref) |  |  |
| Indigenous origin | 0.98  [0.85 – 1.12] | 0.94  [0.80 – 1.11] |
| **Region of residence** |  |  |
| Major city (ref) |  |  |
| Regional city and remote area | 1.03  [0.00 – 1.05] | 0.97  [0.94 – 1.01] |
| **Long–term condition or disability** |  |  |
| No (ref) |  |  |
| Yes | 0.99  [0.99 – 1.02] | 1.01  [1.00 – 1.03] |
| **Smoking status** |  |  |
| Non–smoker (ref) |  |  |
| Current smoker | 0.99  [0.97 – 1.01] | 0.98  [0.96 – 1.00] |
| **Alcohol consumption** |  |  |
| Non–drinker (ref) |  |  |
| Current drinker | 0.99  [0.98 – 1.01] | 1.00  [0.98 – 1.02] |
| **Physical activity** |  |  |
| Less than the recommended level (ref) |  |  |
| Recommended level | 0.99  [0.98 – 1.01] | 0.99  [0.98 – 1.01] |
| **Employment contract** |  |  |
| Employed (ref) |  |  |
| Unemployed | 1.04*  [1.02 – 1.05] | 1.04*  [1.03 – 1.06] |
| Not in labour force | 1.02  [0.99 – 1.05] | 1.03  [1.00 – 1.07] |
| **Family doctor or GP visits (counts)** | 0.99  [1.00 1.00] | 1.00*  [1.00 – 1.00] |
| **Hospital admissions (counts)** | 0.97*  [0.99 – 1.00] | 1.00*  [1.00 – 1.01] |
| **Private health insurance** |  |  |
| No (ref) |  |  |
| Yes | 1.10*  [1.08 – 1.12] | 1.22*  [1.09 – 1.36] |
| Unknown/no response/ not applicable | 1.06*  [1.03 – 1.09] | 1.15*  [1.12 – 1.17] |
| **HRQoL (SF–36 general health)** | 1.00*  [0.99 – 1.00] | 1.00*  [1.00 – 1.00] |
| N | Persons = 20,085  Observations = 41,848 | Persons = 13,719  Observations = 28,593 |

Notes: 1) 95% confidence intervals are reported in parentheses; 2) * Indicate significance at the 5% level; 3) aOR, adjusted odds ratio; Ref, reference category.

Figure A1: Frequency distribution of total household healthcare expenditure.
